# Somatosensory Evoked Field in Response to Visuotactile Stimulation in 3- to 4-Year-Old Children

**DOI:** 10.3389/fnhum.2014.00170

**Published:** 2014-03-24

**Authors:** Gerard B. Remijn, Mitsuru Kikuchi, Kiyomi Shitamichi, Sanae Ueno, Yuko Yoshimura, Kikuko Nagao, Tsunehisa Tsubokawa, Haruyuki Kojima, Haruhiro Higashida, Yoshio Minabe

**Affiliations:** ^1^International Education Center, Kyushu University, Fukuoka, Japan; ^2^Department of Psychiatry and Neurobiology, Graduate School of Medical Science, Kanazawa University, Kanazawa, Japan; ^3^Research Center for Child Mental Development, Kanazawa University, Kanazawa, Japan; ^4^Higher Brain Functions and Autism Research, Department of Child Development, United Graduate School of Child Development, Osaka University, Osaka, Japan; ^5^Department of Anesthesiology, Kanazawa University, Kanazawa, Japan; ^6^Department of Psychology, Kanazawa University, Kanazawa, Japan

**Keywords:** magnetoencephalography, somatosensory evoked field, somatosensory cortex, preschool child, visuotactile stimulation

## Abstract

A child-customized magnetoencephalography system was used to investigate somatosensory evoked field (SEF) in 3- to 4-year-old children. Three stimulus conditions were used in which the children received tactile-only stimulation to their left index finger or visuotactile stimulation. In the two visuotactile conditions, the children received tactile stimulation to their finger while they watched a video of tactile stimulation applied either to someone else’s finger (the finger-touch condition) or to someone else’s toe (the toe-touch condition). The latencies and source strengths of equivalent current dipoles (ECDs) over contralateral (right) somatosensory cortex were analyzed. In the preschoolers who provided valid ECDs, the stimulus conditions induced an early-latency ECD occurring between 60 and 68 ms mainly with an anterior direction. We further identified a middle-latency ECD between 97 and 104 ms, which predominantly had a posterior direction. Finally, initial evidence was found for a late-latency ECD at about 139–151 ms again more often with an anterior direction. Differences were found in the source strengths of the middle-latency ECDs among the stimulus conditions. For the paired comparisons that could be formed, ECD source strength was more pronounced in the finger-touch condition than in the tactile-only and the toe-touch conditions. Although more research is necessary to expand the data set, this suggests that visual information modulated preschool SEF. The finding that ECD source strength was higher when seen and felt touch occurred to the same body part, as compared to a different body part, might further indicate that connectivity between visual and tactile information is indexed in preschool somatosensory cortical activity, already in a somatotopic way.

## Introduction

Magnetoencephalography (MEG) has become an important tool to investigate cortical activity related to sensory or cognitive processing in children of various ages (e.g., Kimura et al., 2004; Chen et al., 2010; Ciesielski et al., 2010; Gummadavelli et al., 2013). Until recently, pediatric MEG has been predominantly performed with systems designed for adult heads. For young children, such as those of preschool age, the adult MEG helmet is not ideal. Preschoolers have considerably smaller heads than adults, and since magnetic field strength decreases with increasing distance between the expected source location and the MEG sensor array (Marinkovic et al., 2004; Gaetz et al., 2008), MEG measurements can be reliably obtained only if the children are repositioned such that one side of their head is as close to the sensor surface as possible (e.g., Pihko et al., 2009). A further requirement is that the children have to minimize head and bodily movements during testing. Under natural testing conditions this is especially challenging for preschoolers aged 2- to 5-years old, since children in this age group are generally less able to suppress movements and to follow procedural instructions (for a review see Pang, 2011). While substantial MEG research has been performed with sleeping or sedated preschoolers in clinical evaluation settings (e.g., Bercovici et al., 2008; Schwartz et al., 2008; Pihko et al., 2009), few studies so far have accomplished preschool MEG measurements under natural and awake testing conditions in an adult MEG system (Fujioka and Ross, 2008; Gaetz et al., 2010).

In order to facilitate pediatric MEG, a system with a child-customized helmet has recently been developed. This helmet allows a more natural fit around the child’s head and has taken away the need for repositioning the head in the dewar (e.g., Gaetz et al., 2008). To date, child-customized MEG has been successfully used with 3- to 6-year-old children to obtain auditory evoked fields to broadband noise (Johnson et al., 2010) and speech (Kikuchi et al., 2011; Ueno et al., 2012; Yoshimura et al., 2012). In the present study, we employed the system to study preschool cortical activity related to modalities other than hearing. We report a study on preschool somatosensory evoked field (SEF) in response to tactile and (multisensory) visuotactile stimuli.

Our first purpose was to expand the literature on preschool SEF. Preschool SEF in response to tactile stimulation has been reported in relatively few studies, each employing an adult MEG helmet (Gondo et al., 2001; Xiang et al., 2004; Gaetz et al., 2008; Pihko et al., 2009). Pihko et al. (2009) found that, during tactile stimulation to the finger, preschool children (1.6- to 6-years old) show a first prominent deflection in the waveform at around 50 ms (M50 component) over contralateral somatosensory cortex. The same study showed that an earlier component at around 30 ms occurs in toddlers and adults, but seldom in preschoolers. A later deflection at around 100 ms has been observed during stimulation to the thumb of toddlers (Gondo et al., 2001). This deflection, however, has not yet been reported in older preschoolers. Since still only few MEG data on preschool SEF exist, here we further investigate the deflections in the MEG waveform in 3- to 4-year-old children in response to tactile stimulation to the left index finger. The children were in natural, awake resting conditions and positioned in a child-customized MEG system.

Our second purpose was to investigate whether the deflections in the preschool waveform to tactile stimulation would already reflect modulation through visual information. In adults, it has been shown that merely watching stimulation to someone else’s body part induces somatosensory activation in the viewer (e.g., Ebisch et al., 2008; Pihko et al., 2010; Meyer et al., 2011; for a review see Keysers et al., 2010). Modulatory effects of visual information containing “touch” to someone else’s leg (Keysers et al., 2004), face, neck (Blakemore et al., 2005), and hand (Pihko et al., 2010) have been reported. Modulatory effects of vision have also been reported on SEF in response to tactile stimulation to an observer who watched tactile stimulation to others at the same time (Schaefer et al., 2006).

Some studies with adults have suggested that visuotactile brain responses are somatotopically organized. For example, in a study without direct tactile stimulation to the observer, Blakemore et al. (2005) found that the head area of primary somatosensory cortex was activated when observers watched touch to the face, but not to the neck. Other support for somatotopic organization has been reported in the field of action observation. Both the execution of hand and mouth actions and the mere observation of others’ hand and mouth actions evoked activity in the corresponding premotor areas (Gazzola et al., 2006). Also in studies in which an observer watched stimulation to others while receiving direct tactile stimulation to the self, adult somatosensory activity seemed to reflect topographic selectivity. Motor-evoked potentials recorded from a hand region were differently modulated during observation of painful stimuli to the same hand region as compared to the foot (Avenanti et al., 2005). In preschoolers, however, modulation of somatosensory responses through visual information has not yet been explored.

**Figure 1:**
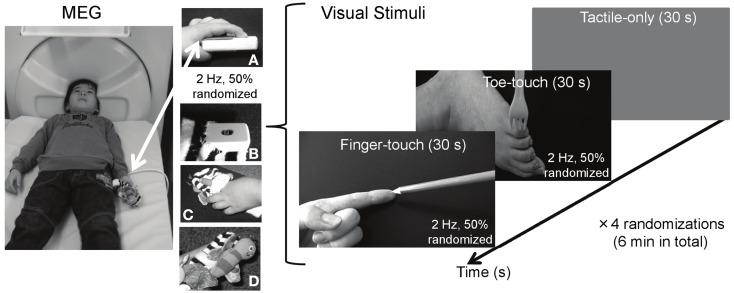
**Visuotactile stimulation used during MEG measurements of preschool somatosensory cortex**. While tactile stimulation was applied to the left index finger, 3- to 4-year-old children watched tactile stimulation to someone else’s left index finger (finger-touch condition) or to someone else’s toe (toe-touch condition). The tactile stimulation to the children’s left index finger during MEG measurements was applied through a piezo-electric stimulator **(A)**, decorated with a butterfly-like stuffed doll [**(B)** top-view; **(C)** top-view with child’s finger; **(D)** side-view].

To this end, we studied the effect of vision on preschool SEF with visuotactile stimuli that either concerned the same or a different body part. The stimuli comprised two visuotactile conditions (Figure 1). While the children received tactile stimulation to their own left index finger, they either watched mild stimulation of the left index finger (finger-touch condition) or the left toe (toe-touch condition) of someone else on a video. Besides expanding the data set on preschool SEF to unimodal tactile stimulation, i.e., without any visually presented information, we investigated whether already at the preschool age SEF undergoes modulation through vision that is somatotopically selective. If so, the adult data would suggest that SEF source strength differs between stimulation with congruent visuotactile information (finger-touch condition) and incongruent visuotactile information (toe-touch condition).

## Materials and Methods

### Ethics statement

The procedures were approved by the Ethics Committee of Kanazawa University Hospital and followed the Declaration of Helsinki. At least one parent/caregiver of each preschool participant provided written, informed consent before participation. Face-scale ratings obtained after the experiment (see below) indicated that none of the preschoolers were uncomfortable with the tactile stimulation seen on video and that felt on the finger.

### Participants

Thirty-eight Japanese preschool children (22 females and 16 males), with an average and median age of 4 years ± 1 month, participated in the experiment. All were children from staff members of Kanazawa University Hospital or recruited from nursery schools near the hospital, in Kanazawa city, Japan. The preschoolers were right-handed, as reported by their parent(s)/caregiver(s). All had normal vision and were in normal physical health. Test results from the Japanese adaptation of the Kaufman Assessment Battery for Children (Kaufman et al., 1987) indicated that all were typically developing.

### Tactile stimulus and visuotactile stimuli

Tactile stimulation was delivered to the infant’s left index finger. A 100-Hz waveform, generated by a sinusoidal oscillator (Uchida Electric, Tokyo, Japan), was used to drive a piezo-electric pulse generator attached to the finger. The repetitive pulse caused a displacement of approximately 0.5 mm of the finger tissue. Each pulse lasted 4 ms and was presented at 2 Hz, with an average time randomization of 50%, for a period of 6 min in total. The intensity of the tactile stimulation was kept constant across participants. The pulse generator and the upper part of the magic tape used to hold the child’s finger over the pulse were decorated with a miniature stuffed butterfly made of soft cloth. This was wrapped gently around the participant’s index finger.

Two visual stimulus conditions with a duration of 30 s were used (Figure 1). In the finger-touch condition, the infant watched a female left hand set against a black background (5.3 cd/m^2^). The hand’s index finger was alternately and repetitively touched by one of the following six pointy objects: a tine of a metal fork, a tine of a plastic yellow fork, the tip of a black pen, and the tip of a blue pencil, a red pencil, or a yellow pencil. Visual objects were changed as to avoid the theoretical chance of interference from visually evoked magnetic field induced by too much repetition of the same visual stimulus. Movement lasted 5 s for each of the six objects, together constituting a finger-touch stimulus of 30 s. The order of the objects was randomized for each preschool child. In the toe-touch condition, the participant watched a female left toe against the same black background. In a similar vein as in the finger-touch condition, in six randomized series of 5 s, the outside of the foot just below the little toe was touched by one of the six objects. In both the finger- and the toe-touch conditions, the objects touched the human tissue with a frequency of 2 Hz, with successive touches randomly occurring within 250–750 ms after one another. Since the felt and observed stimulation occurred to the same body part in the finger-touch condition, tactile and seen stimulation were desynchronized in order to avoid the illusion that the observed body part (e.g., the finger in the finger-touch video) was part of the observer’ own body. Such a complex percept would be difficult to study reliably with preschoolers and might have been confusing to them. The two visuotactile conditions were presented with a tactile-only condition, in which the infant watched a gray screen (26.5 cd/m^2^) with a white fixation cross to rest his/her eyes. The fixation cross subtended 1 × 1 deg in visual angle (112.4 cd/m^2^) and was centered in the middle of the screen.

The finger-touch, toe-touch, and the tactile-only displays were generated and controlled by a personal computer (NEC VersaPro VA9), and back-projected from a display projector (Sharp PG-B10S) through four mirrors onto a 30 cm × 21 cm screen, viewed from supine position through a mirror attached to the MEG dewar. The three stimulus conditions were each repeated four times, with the order randomized for each infant. In total, 240 visual events in the finger-touch and toe-touch conditions were displayed. The total duration of the MEG measurements was 6 min. The infant was instructed to remain in a fixed bodily position and watch the screen.

### Stimuli (dis)comfort judgments

Before MEG measurement, the infant was asked whether the tactile stimulation to his/her index finger was comfortable or not and told that MEG-recording could be abandoned any time he/she wanted. None of the preschoolers reported dislike or discomfort toward the piezo-electric stimulator and/or the tactile stimulation, which was reported as mild, painless stimulation above sensation threshold. None of the preschoolers opted out of the experiment. After the experiment a face-scale (Wong and Baker, 1988) was used to obtain subjective impressions of the infant’s (dis)comfort with the tactile stimulation to the index finger and that seen on video. The face-scale consisted of pictures of cartoon-like faces, showing happiness (smiling) or sadness (in tears) in five intermediate steps (0–4), with “0” representing “no sensation” to “4” representing “uncomfortable sensation.” When asked what their score would be if a child were to receive an injection, all preschoolers responded the maximum “4.” This indicated they had understood the usage and range of the face-scale. Face-scale ratings were obtained from 35 out of 38 infants. Overall, their ratings showed that the stimuli were not discomforting. In the tactile-only condition, (dis)comfort to the finger stimulation was judged as 1.34 ± 0.26. When watching the finger-touch and the toe-touch video, this was 1.51 ± 0.23 and 1.30 ± 0.21, respectively. One-way analysis of variance (ANOVA) showed no significant difference between conditions [*F*(2, 68) = 0.28, *p* = 0.75].

### MEG measurements and data analysis

Somatosensory evoked field was recorded with a 151-channel SQUID (Super conducting Quantum Interference Device) whole-head coaxial gradiometer MEG system for children (PQ1151R, Yokogawa Electric, Kanazawa, Japan). The pick-up coils of the MEG system were 15.5 mm in diameter, the mean distance between two adjacent coils was 22 mm, and the cool-to-warm (dewar-coil) separation was 20 mm. Recordings were made in a three-layered, magnetically shielded room (Daido Steel, Nagoya, Japan), installed at the MEG-research center of Yokogawa Electric Corporation (Kanazawa, Japan). In an attempt to make the measurement environment less intimidating to the infants, the shielded room was decorated with colorful pictures of cartoon characters, familiar and liked by most Japanese preschoolers. The infant lay in a supine position on a tray-bed (Yokogawa, PQ11TA) adjusted to the height of the MEG dewar. One staff member (author YY) stayed in the shielded room to comfort the child and to encourage him/her to maintain a steady bodily position.

Magnetoencephalography data were acquired with a sampling rate of 1000 Hz and filtered with a 200-Hz low-pass filter. Time series were segmented into windows of 250 ms (−50 to 200 ms). Around 195–214 segments were averaged for each of the 151 magnetic sensors after baseline correction (−30 to −10 ms). An average of 9.7% of the segments with a noise contamination exceeding ±4 pT was excluded from the data before principal component analysis was performed for general noise reduction. At least 195 tactile events were analyzed for each condition. We determined the position of the head within the MEG dewar by measuring the magnetic fields after passing currents through coils that were attached at three locations of the head surface as the fiduciary points, with respect to the landmarks (nasion and pre-auricular points or mastoid tips). Since magnetic resonance imaging (MRI) anatomical data of the infants were not obtained, a 3-D coordinate system based on fixed MEG sensor locations was applied to calculate the equivalent current dipoles (ECDs) by using a spherical model of the volume conductor. This was fitted to the center of the fixed MEG coordinate system, after confirmation that each infant’s head was located in the center of MEG dewar, by measuring the three locations of the head surface mentioned above (also see Yoshimura et al., 2012).

The single ECD model (Sarvas, 1987) was used to estimate the “center of gravity” of the current sources. We analyzed the latencies and the number of major deflection(s) in the waveform in subsequent order, and considered ECDs as valid only when (i) goodness of fit (GOF) was over 80%; (ii) the location of estimated dipoles was stable within ±5 mm of each coordinate during a period of at least 6 ms; and (iii) dipole intensities were less than 80 nA/m. As a consequence of following these criteria, there were cases in which only a single ECD in a multiple-peak waveform was considered for further analyses of source strength. ECDs for each stimulus condition were categorized according to latency with k-means cluster analysis, with the number of clusters set at three, according to the maximum number of observed major deflections in the data. Statistical analyses on the source strength were performed after taking the natural logarithm. Distribution normality was tested with the Shapiro–Wilk test and variance homogeneity was tested with Levene’s test. If normality and variance homogeneity were not violated, analysis of variance (ANOVA) with independent samples was used to test the source strength between ECD latency categories within stimulus conditions, and between similar latency categories of different stimulus conditions. Paired *t*-tests were also performed to test source strengths between similar latency categories of different stimulus conditions, with Bonferroni correction on the alpha-level. Predominance of ECD direction within each latency category (anterior or posterior) was tested with the binomial test.

## Results

### Tactile-Only condition

**Figure 2:**
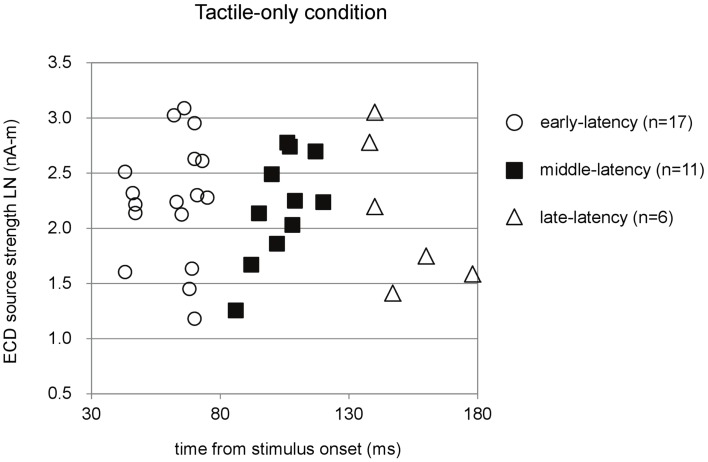
**Preschool ECD latencies and source strengths in the tactile-only condition**. ECDs were obtained during piezo-electric stimulation to the left index finger of 3- to 4-year-old children. Note that the same participant could have provided data for more than one latency category, i.e., showed double-peak or triple-peak deflections in the MEG waveform that obeyed the ECD criteria. LN, natural logarithm; nA-m, nano-Ampere per meter; ms, milliseconds.

Figure 2 shows the three latency categories of the major deflections in the waveforms, with corresponding ECD source strengths, obtained in the tactile-only condition. The latencies of the ECDs that were in accordance with the criteria fell into categories that from here on are referred to as early-, middle-, and late-latency deflections. In the data of 17 infants, an early major deflection occurred on average at 61.65 ± 2.78 ms. In the waveform of four infants, this early peak was the only deflection observed. In eight infants, it was the first of a double-peak waveform, while in five infants, it was the first of a triple-peak waveform. In the waveforms of 11 infants, a middle-latency deflection could be observed on average at 103.82 ± 3.09 ms. In four infants, this middle-latency peak was the only deflection in the waveform. In three infants, it was the second deflection in a double-peak waveform, while in the remaining four, it was the second in a triple-peak waveform. In six infants, a late deflection in the waveform occurred on average at 150.50 ± 6.42 ms. In two infants, it was the late second in a double-peak waveform, whereas in four infants, it was the last in a triple-peak waveform.

**Figure 3:**
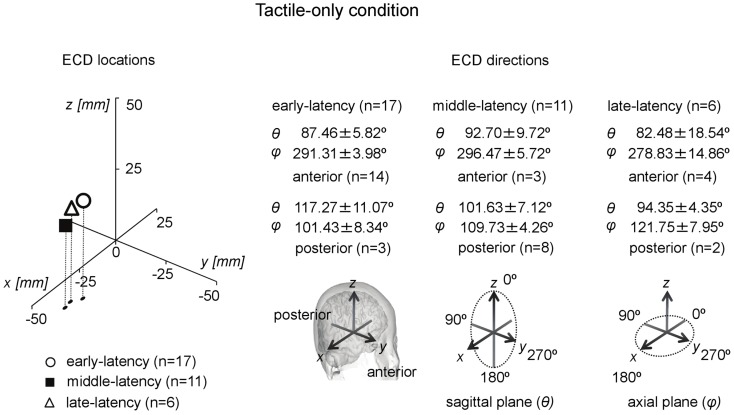
**Preschool ECD locations and directions in the tactile-only condition**. ECDs were obtained during tactile stimulation to the left index finger, resulting in ECD locations in contralateral right hemisphere. Early-latency ECDs predominantly had an anterior direction. No significantly dominant direction pattern was observed for middle-latency and late-latency ECDs. Note that the same participant could have provided data for more than one category, i.e., showed double-peak or triple-peak deflections in the MEG waveform that obeyed the ECD criteria.

In total, only five infants provided waveforms with double-peak deflections both of which obeyed the ECD criteria, whereas only four cases were found in which all three ECDs in a triple-peak waveform were valid. Paired comparisons between ECD source strengths over two or three latency categories would therefore suffer from a lack of power. Instead of repeated-measures ANOVA, one-way ANOVA for independent samples was performed between the source strengths of the ECDs in the three latency categories. Shapiro–Wilk tests showed no evidence for non-normality for the early-latency (df = 17, *p* = 0.49), middle-latency (df = 11, *p* = 0.61), and the late-latency ECDs (df = 6, *p* = 0.47), and homogeneity of variance was met as well (Levene statistic = 0.63, *p* = 0.54). The ANOVA showed that source strength did not significantly differ with latency category in the tactile-only condition [*F*(2, 33) = 0.12, *p* = 0.89]. The dipole coordinates and directions of the valid ECDs in the tactile-only condition are depicted in Figure 3. The tactile stimulation of the left index finger induced ECDs that were located over contralateral (right) cortex, approximately over somatosensory areas. Because of the young age of the participants, MRI-plots were not performed. Early-latency ECDs predominantly had an anterior dipole direction (14 out of 17 ECDs), rather than a posterior dipole direction. The binomial test showed that this difference was significant (two-sided, *p* < 0.05). Middle-latency ECDs were more often posteriorly directed (8 out of 11 ECDs), while late-latency ECDs were anteriorly directed in two-thirds of the cases (4 out of 6 ECDs). These trends in ECD direction in the middle-latency and late-latency category were not significant.

### Visuotactile conditions

The finger-touch condition induced major deflections with latency clusters that were similar as those observed in the tactile-only condition. In the finger-touch condition, an early-latency deflection occurred at 67.44 ± 1.60 ms (*n* = 16). In five children, this was the only major deflection in the waveform. In five children, it was the first of a double-peak waveform, and in six children, it was the first of a triple-peak waveform. A middle-latency deflection occurred at 101.50 ± 2.45 ms (*n* = 16). In four children, this was the only deflection in the waveform, in seven children, it was the second in a double-peak waveform, and in five children, it was the second in a triple-peak waveform. A late-latency deflection in the finger-touch condition occurred on average at 139.70 ± 3.38 ms (*n* = 10). It was the only deflection in the waveform of two children, the late second in the waveform of three children, and the third in the triple-peak waveform of five children. Only six children provided valid ECDs for both the early- and middle-latency categories, whereas only four children provided waveforms in which all ECDs in a triple-peak waveform were valid. One-way ANOVA for independent samples was therefore performed also for the finger-touch data. Shapiro–Wilk tests showed no normality violations for the early-latency (df = 16, *p* = 0.26), middle-latency (df = 16, *p* = 0.06), and the late-latency ECD groups (df = 10, *p* = 0.91). The variances among the three groups also did not differ significantly (Levene statistic = 0.42, *p* = 0.66). One-way ANOVA showed no significant difference in source strength between the latency categories in the finger-touch condition [*F*(2, 41) = 0.24, *p* = 0.79].

In the toe-touch condition an early-latency deflection occurred at 60.82 ± 2.53 ms (*n* = 22). This deflection was the only peak in the waveform of six children, the first in a double-peak waveform observed in 12 children, and the first in a triple-peak waveform of four children. A middle-latency deflection occurred on average at 97.75 ± 2.75 ms (*n* = 12). This was a single deflection in the waveform of seven children, the second in a double-peak waveform in two children, and the second in a triple-peak waveform in three children. A late-latency deflection in the toe-touch condition appeared at 139.27 ± 3.88 ms (*n* = 15). For two children, this was the only valid major deflection. For eight children, it was the second deflection in a double-peak waveform, and for five children, it was the third in a triple-peak waveform. Four children provided valid ECDs for both the early- and middle-latency categories, and only three preschoolers provided waveforms in which all ECDs in a triple-peak waveform were valid. Shapiro–Wilk tests showed no normality violations for the early-latency (df = 22, *p* = 0.09), middle-latency (df = 12, *p* = 0.53), and the late-latency ECD groups (df = 15, *p* = 0.21). The variances among the three groups also did not differ significantly (Levene statistic = 0.48, *p* = 0.62). One-way ANOVA for independent samples showed that source strength differed significantly between latency categories in the toe-touch condition [*F*(2, 48) = 3.59, *p* = 0.036]. *Post hoc* Bonferroni tests showed that for the toe-touch condition the source strength in the early-latency category was higher than that in the middle-latency category (*p* = 0.034).

The ECDs in all three latency categories in both visuotactile conditions were located at the contralateral hemisphere. Analysis of ECD directions showed that ECDs in the early-latency category had a predominantly anterior direction. In the finger-touch condition 16 out of 17 ECDs were anteriorly directed. The binomial test showed that this difference was significant (two-sided, *p* < 0.01). In the toe-touch condition, 18 out of 22 ECDs with an early-latency had an anterior direction, which was significant as well (two-sided, *p* < 0.01). ECDs in the middle-latency category were significantly more posteriorly than anteriorly directed. In the finger-touch condition, 14 out of 16 ECDs (two-sided, *p* < 0.01), and in the toe-touch condition, 10 out of 12 (two-sided, *p* < 0.05) were posteriorly located. Late-latency ECDs again were significantly more anteriorly directed. Nine out of 10 ECDs in the finger-touch condition and 12 out of 15 ECDs in the toe-touch condition had an anterior direction (two-sided, *p* < 0.05 in both cases).

### Comparisons between ECD source strengths in the tactile-only and the visuotactile conditions

For each latency category, ECD source strength between the three stimulus conditions was first compared with ANOVA for independent samples. ANOVA was performed after Levene’s tests showed no deviance from distribution normality in the early-latency (Levene’s statistic = 0.63, *p* = 0.53), the middle-latency (Levene’s statistic = 1.38, *p* = 0.37), and the late-latency (Levene’s statistic = 1.16, *p* = 0.33) ECD source strengths between stimulus conditions. In the case of unpaired comparisons, source strength between the tactile-only, the finger-touch, and the toe-touch condition did not differ for the early-latency [*F*(2, 54) = 0.12, *p* = 0.89], the middle-latency [*F*(2, 38) = 1.35, *p* = 0.27], and the late-latency [*F*(2, 30) = 0.18, *p* = 0.83] categories.

**Figure 4:**
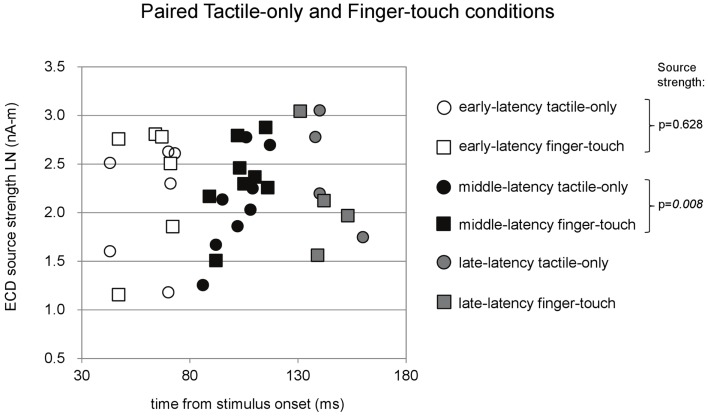
**Preschool ECD latencies and source strengths in paired tactile-only and finger-touch conditions**. Circles show the tactile-only data and squares show the finger-touch data. White symbols show early-latency pairs (*n* = 6), black symbols show middle-latency pairs (*n* = 8), and gray symbols show late-latency pairs (*n* = 4). In the middle-latency category, ECD source strength was significantly higher in the finger-touch (filled black squares) than in the tactile-only condition (filled black circles). LN, natural logarithm; nA-m, nano-Ampere per meter; ms, millisecond.

**Figure 5:**
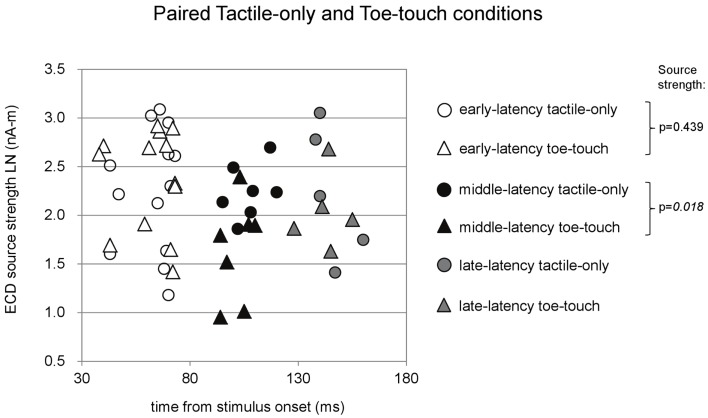
**Preschool ECD latencies and source strengths in paired tactile-only and toe-touch conditions**. Circles show the tactile-only data and triangles show the toe-touch data. White symbols show early-latency pairs (*n* = 13), black symbols show middle-latency pairs (*n* = 7), and gray symbols show late-latency pairs (*n* = 5). In the middle-latency category, ECD source strength was higher in the tactile-only (filled black circles) than in the toe-touch condition (filled black triangles), but the difference strictly did not reach significance with Bonferroni correction of the alpha-level. LN, natural logarithm; nA-m, nano-Ampere per meter; ms, millisecond.

**Figure 6:**
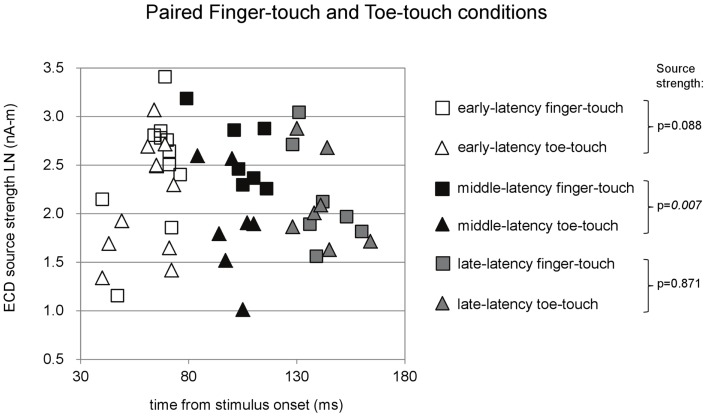
**Preschool ECD latencies and source strengths in paired finger-touch and toe-touch conditions**. Squares show the finger-touch data and triangles show the toe-touch data. White symbols show early-latency pairs (*n* = 11), black symbols show middle-latency pairs (*n* = 7), and gray symbols show late-latency pairs (*n* = 7). In the middle-latency category, ECD source strength was significantly higher in the finger-touch (filled black squares) than in the toe-touch condition (filled black triangles). LN, natural logarithm; nA-m, nano-Ampere per meter; ms, millisecond.

Paired comparisons between ECD source strengths in similar latency categories could be made with a limited number of cases. Because few children provided data for all three latency categories in all three stimulus conditions, repeated-measures ANOVA was not performed. Instead, where possible, we performed *t*-tests between pairs of stimulus conditions and applied Bonferroni correction on the alpha-level. The main results are depicted in Figures 4–6. Paired comparisons between the tactile-only and the finger-touch condition were made for the early-latency and the middle-latency category (Figure 4). The late-latency category provided only four pairs and thus was not tested. Six children provided valid pairs of early-latency ECD source strengths. Shapiro–Wilk tests showed no violations of distribution normality for the early-latency tactile-only (df = 6, *p* = 0.11) and the finger-touch condition (df = 6, *p* = 0.06). Under similar variances (Levene’s statistic = 0.05, *p* = 0.83), ECD source strength did not differ between the two stimulus conditions (*t* = −0.52, df = 5, *p* = 0.63). Eight children provided valid ECDs in the middle-latency category. Both the data in the tactile-only (df = 8, *p* = 0.89) and the finger-touch conditions (df = 8, *p* = 0.40) were normally distributed and showed similar variances (Levene’s statistic = 0.43, *p* = 0.53). In the middle-latency category, ECD source strength in the finger-touch condition was significantly higher than in the tactile-only condition with a Bonferroni-corrected alpha-level of 0.017 for multiple paired comparisons (*t* = −3.66, df = 7, *p* < 0.01).

Paired comparisons between the tactile-only and the toe-touch conditions were also made just for the early-latency and the middle-latency category, since late-latency data were provided by only five children (Figure 5). Thirteen children provided valid pairs of early-latency ECD source strengths, which were normally distributed in the tactile-only condition (df = 13, *p* = 0.46) and the toe-touch condition (df = 13, *p* = 0.06). Under similar variances (Levene’s statistic = 0.34, *p* = 0.57), no significant difference was found between early-latency source strengths (*t* = −0.80, df = 12, *p* = 0.44). Seven pairs could be formed with valid middle-latency ECD data. These were normally distributed in both the tactile-only condition (df = 7, *p* = 0.93) and the toe-touch condition (df = 7, *p* = 0.53) and showed no unequal variances (Levene’s statistic = 2.92, *p* = 0.11). In the middle-latency category, ECD source strength in the tactile-only condition was higher than that in the toe-touch condition (*t* = 3.23, df = 6, *p* = 0.018). With the Bonferroni-corrected alpha-level (*p* = 0.017), however, this difference would strictly be not significant.

**Figure 7:**
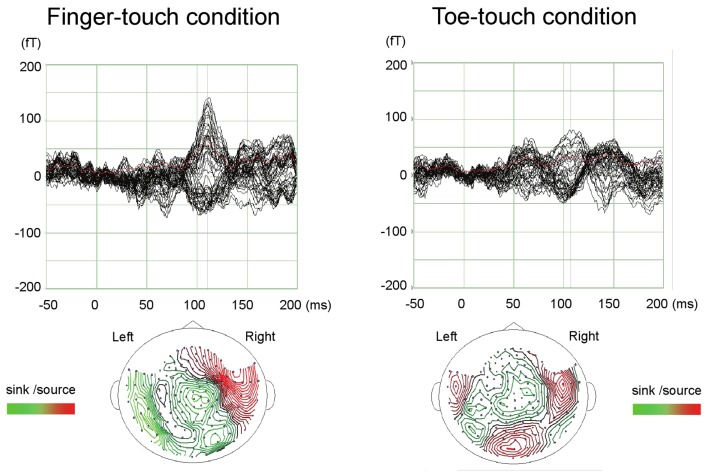
**An example of preschool SEF waveforms in the visuotactile finger-touch condition (left) and the toe-touch condition (right)**. The waveforms (top) and estimated ECDs (bottom) are shown for a 36-month-old girl. fT, femto-Tesla.

**Figure 8:**
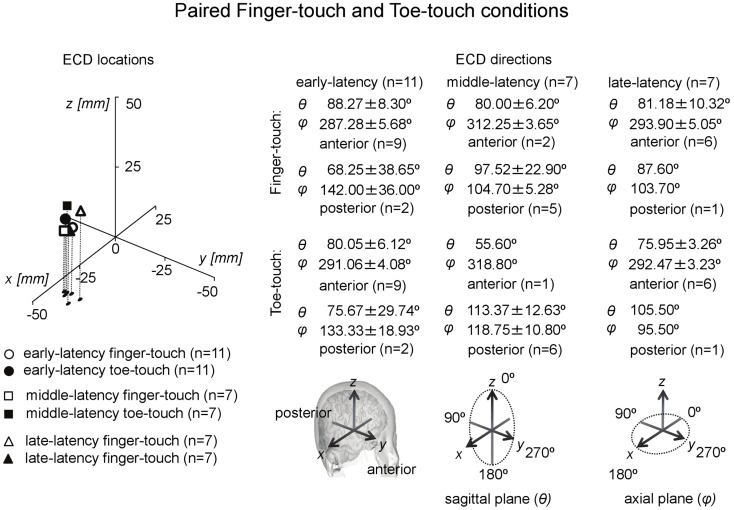
**Preschool ECD locations and directions in paired finger-touch and toe-touch conditions**. ECDs were obtained during tactile stimulation to the left index finger, while the children watched a video of tactile stimulation to someone else’s finger (finger-touch) or toe (toe-touch). Note that the same participant could have provided data for more than one category, i.e., showed double-peak or triple-peak deflections in the MEG waveform that obeyed the ECD criteria.

Paired comparisons between the two visuotactile conditions could be made for all three latency categories (Figure 6). In the early-latency category 11 paired comparisons could be made. Shapiro–Wilk tests showed no violation of source strength normality in the early-latency finger-touch (df = 11, *p* = 0.34) and toe-touch condition (df = 11, *p* = 0.48). The variances in the source strengths also did not differ significantly (Levene statistic = 0.30, *p* = 0.59), and neither did the source strengths themselves (*t* = 1.89, df = 10, *p* = 0.09). For seven pairs in the late-latency category, source strength normality in the finger-touch (df = 7, 0.34) and the toe-touch (df = 7, 0.25) condition was not violated. Under homogeneity of variances (Levene’s statistic = 0.06, *p* = 0.81), source strength in the late-latency categories did not significantly differ between the finger-touch and the toe-touch conditions (*t* = 0.17, df = 6, *p* = 0.87). Also for the middle-latency category seven pairs could be made. Both the source strength in the finger-touch (df = 7, *p* = 0.24) and toe-touch conditions (df = 7, *p* = 0.55) was normally distributed and variances were homogeneous (Levene’s statistic = 0.33, *p* = 0.57). The middle-latency source strength observed in the finger-touch condition was significantly higher than that in the toe-touch condition (*t* = 3.97, df = 6, *p* < 0.01). An example of the difference in the waveforms induced by the finger-touch and toe-touch conditions is depicted in Figure 7. ECD locations and directions of the paired finger- and toe-touch conditions are shown in Figure 8.

In summary, comparisons between ECD source strengths observed in the middle-latency categories of the three stimulus conditions showed that source strength was higher in the tactile-only condition than in the toe-touch condition. With a decreased alpha-level of 0.017 to correct for multiple comparisons, however, this difference was not significant. Even with alpha-level correction, however, middle-latency ECD source strength in the finger-touch condition was significantly higher than in the toe-touch and the tactile-only conditions.

## Discussion

The present study is the first to use a child-customized MEG system to study somatosensory responses in preschool children. Besides a tactile-only condition, in which the left index finger of the preschoolers was stimulated, two visuotactile conditions were used. In one condition, the child received tactile stimulation to the index finger and at the same time watched a video of someone else being touched at the index finger. In another condition, the child received the tactile stimulation to the finger while watching a video of someone else being touched on the toe. ECD analysis showed that all three conditions induced contralateral (right-hemispheric) activity, which enabled valid dipole estimation in about 60% of the children. For all three stimulus conditions, a first valid ECD could be identified with a latency between 60 and 68 ms. This early-latency ECD had a predominantly anterior direction. The early-latency ECD strongly resembles that reported by Pihko et al. (2009), who analyzed preschool SEF with an adult MEG system. They too found a major deflection in the preschool waveform associated with an anteriorly directed dipole occurring around 60 ms (M60), but mainly in toddlers around 1 year of age in combination with an earlier component around 30 ms (M30). According to the authors, few preschoolers in the age of 1.6- to 6-years of age showed the M30, which is in accordance with the present study, but the preschoolers in the study of Pihko et al. (2009) did show a relatively prominent adult-like M50 with a posterior dipole direction. In the present study, over all conditions combined and including the cases that could not be considered in the paired comparisons, only eight preschoolers showed a posterior early-latency ECD. The average latency of this ECD was 50 ± 3.86 ms and thus indeed an M50. The vast majority (48) of the combined early-latency ECDs, however, had an anterior location with a longer latency between 60 and 68 ms. In the present study, most preschoolers thus still showed an M60. The source strengths of these early-latency ECDs did not differ between stimulus conditions. In the toe-touch condition, the early-latency ECD had a significantly more pronounced source strength than the following, middle-latency ECD.

The second, middle-latency ECD occurred on average between 97 and 104 ms in all three stimulus conditions. This latency seems to correspond with the data of Gondo et al. (2001), who reported a deflection with a latency of about 100 ms in the SEF of toddlers in response to tactile stimulation to the thumb. In the present data, the middle-latency deflection was posteriorly directed in 32 out of 39 ECDs combined over the three stimulus conditions. We further found that the source strength of the middle-latency ECD was subject to visual modulation. Although not significant with unpaired comparisons, the middle-latency ECD source strength in the finger-touch condition was significantly higher than that in the toe-touch condition in the case of paired comparisons (*n* = 7). Although paired comparisons between the source strengths observed in the visuotactile and tactile-only conditions might be inappropriate, since the tactile-only condition was a condition in which the participants could rest their eyes on the screen and arguably made less eye movements, we further found that the finger-touch condition induced a significantly higher middle-latency source strength than the tactile-only condition for *n* = 8. ECD source strength, by contrast, was higher in the tactile-only than in the toe-touch condition in the middle-latency category. With Bonferroni correction on the alpha-level, however, this difference was not significant.

The differences in middle-latency source strength between stimulus conditions suggest that visual information modulates preschool SEF. The difference between congruent (finger-touch) and incongruent (toe-touch) visuotactile stimulation might further suggest that somatotopic linkage for seen and felt touch already develops in early childhood. Out of behavioral necessity, children must learn to recognize congruent visual and tactile information that is behaviorally relevant to them as soon as possible. For example, they must quickly learn to recognize whether an object can cause comfort or pain – often by visuotactile inspection. The sparse literature related to somatotopy in child cortex concerns studies on phantom limb experiences in persons with congenitally absent limbs. In spite of being limb-deficient from birth, some of these persons experienced phantom limbs since early childhood (Poeck, 1964). The representations are likely built up through visuotactile input (Hunter et al., 2003), for example, from observation and feeling the intact limb of the self and others. Further research is necessary, though, to gain more evidence for somatotopy in the preschool brain and to clarify the mechanisms that mediate it. In the present experiment, factors such as attentional engagement toward the stimuli may have contributed to the source strength difference in the paired middle-latency ECDs obtained in the visuotactile conditions. Some MEG studies with visuotactile stimuli have implicated or specifically investigated the role of attentional engagement to the stimuli (Mima et al., 1998; Iguchi et al., 2002, 2005; Hesse et al., 2010). We can speculate, for example, that while watching the finger-touch video the children increased their attention to the stimulation to their own finger. When watching the toe-touch video, however, the children might have “ignored” the stimulation to their finger by concentrating more on their toe. This could have caused a contrast in the response strength between the toe-touch and the finger-touch conditions, assuming that the videos of stimulation to someone else’s body part indeed could manipulate attentional focus of the preschooler to his/her own body part. Future research might attempt to further investigate this by quantifying the viewers’ looking behavior to the video by using an eye-tracking device. Inquiries about attentional engagement to the videos and their own body part might be performed with an interview, although preschoolers might not always provide accurate and reliable answers.

Besides the early- and middle-latency ECDs, a valid ECD with a late-latency could be observed in all the stimulus conditions. The late-latency ECD occurred between 139 and 151 ms and, to our knowledge, has not been reported in preschoolers before. In the tactile-only condition, the dipole direction was anterior in four out of six children. In the two visuotactile conditions this trend was stronger: in 21 out of 25 combined ECDs the dipole direction was anteriorly directed. Significant source strength differences in the late-latency ECDs were not found between stimulus conditions.

In summary, the present study with the child-customized MEG system confirmed the occurrence of an early-latency ECD connected with tactile stimulation to the finger with a latency of about 65 ms and a predominantly anterior direction. Middle-latency ECDs were observed at around 100 ms with a predominantly posterior dipole direction. Source strength differences between paired middle-latency ECDs suggest SEF modulation through visual information in general, with congruent visuotactile information (finger-touch condition) inducing a significantly larger source strength than incongruent visuotactile information (toe-touch condition). This might reflect the development of brain functional connectivity between visual and somatosensory areas, presumably in a somatotopic way. The present preschool data further indicate the occurrence of a late-latency ECD (around 145 ms), which tended to have an anterior direction. Further research on somatosensory activity in preschool cortex is necessary to test the existence and development of (somatotopic) modulation by visual information and to expand the data in general. Also with the child-customized MEG system used here, data contamination due to motion and concentration artifacts limited the quality of the data and, hence, the number of valid ECDs that could be used for statistical comparisons.

## Conflict of Interest Statement

The authors declare that the research was conducted in the absence of any commercial or financial relationships that could be construed as a potential conflict of interest.
